# Serum epidermal growth factor-like domain 7 serves as a novel diagnostic marker for early hepatocellular carcinoma

**DOI:** 10.1186/s12885-021-08491-3

**Published:** 2021-07-03

**Authors:** Meng-Yuan Yang, Fan Wu, Feng Fang, Hao Yang, Jing-Fan Zhang, Guo-Dong Chen, Lian-Yue Yang

**Affiliations:** 1 Department of Surgery, Liver Cancer Laboratory, Xiangya Hospital, Central South University, Changsha, 410008 Hunan China; 2 Department of Gynecology and Obstetrics, The Second Xiangya Hospital, Central South University, Changsha, 410011 Hunan China

**Keywords:** Hepatocellular carcinoma, Eepidermal growth factor-like domain 7, Alpha-fetoprotein, Early diagnosis, Serum marker

## Abstract

**Background:**

Epidermal growth factor-like domain 7 (Egfl7), a recently identified secreted protein, was significantly increased in patients with HCC by our previous studies. However, its efficacy in the diagnosis of early HCC remains unknown. In this study, we therefore evaluate the efficacy of serum Egfl7 for early HCC diagnosis and compare it with alpha-fetoprotein (AFP).

**Methods:**

Serum Egfl7 levels in testing cohort (1081 participants) and validation cohort (476 participants) were measured by a sandwich enzyme-linked immunoassay (ELISA). The cut-off value of Egfl7 was determined by Youden’s index and the efficacies of Egfl7 and AFP in diagnosing early HCC were estimated by receiver operating characteristic (ROC).

**Results:**

Serum Egfl7 was significantly elevated in patients with early HCC than all non-HCC controls in whatever Testing Cohort or Validation Cohort. In the Testing Cohort, ROC curves showed the optimum cut-off value of Egfl7 was 2610 ng/mL and Egfl7 showed a significantly higher sensitivity than AFP in discriminating early HCC from healthy individuals (77.4% vs. 65.3%, *P* = 0.0013) but the area under ROC (AUROC) and accuracy of Egfl7 and AFP were similar (0.860 vs. 0.868, *P* = 0.704; 80.2% vs. 83.8%, *P* = 0.184). In distinguishing patients with early HCC from patients with chronic liver disease (CLD), the AUROC, sensitivity, specificity and accuracy of Egfl7 were 0.800, 75.2, 71.7 and 73.5%, which were all significantly higher than AFP (0.675, 61.8, 62.0 and 61.9% in order). Egfl7 also showed a significant higher sensitivity and accuracy than AFP (76.6% vs. 64.0%, *P* = 0.0031; 79.9% vs. 66.1%, *P* < 0.0001) in differentiating early HCC patients from non-HCC individuals. Additionally, 70.8% of early HCC patients with negative AFP could be diagnosed by Egfl7 and the combined use of Egfl7 and AFP increased the sensitivity to 91.0%. These results were confirmed by a validation cohort.

**Conclusion:**

Egfl7 is a valuable serum marker in the diagnosis of early HCC and could complement the efficacy of AFP, especially in distinguishing early HCC from CLD and identifying patients with AFP-negative early HCC.

## Background

Hepatocellular carcinoma (HCC) is the sixth most common cancer worldwide and the second leading cause of male cancer death in developing countries, which resulting in about 782,000 global deaths each year, while China alone accounting for about 50% [1, 2]. Because of the large population of patients with hepatitis B- or C-related liver cirrhosis, the major risk factor for HCC especially in China, the incidence of HCC will increase over the next decade [1, 3, 4]. Despite the great improvements in treatments, the long-term survival of patients with HCC remains unsatisfactory, with a 5-year survival rate of less than 20%, mainly due to limited effective therapeutic options for HCC patients with late stage [5]. Therefore, the early detection of HCC, providing the opportunity for radical treatment, is crucial to reduce the mortality and improve the long-term prognosis of this lethal malignancy [6].

Currently, basing the limited early diagnostic modalities available, only 30 to 40% of patients with HCC could receive curative treatments [7]. The most widely- used early modalities for clinical diagnosis were ultrasound (US) and serum alpha- fetoprotein (AFP). The reported sensitivity and specificity of US for HCC ≥ 1 cm in diameter were 70 and 48%, respectively [8]. However, when HCCs were less than 1 cm in diameter, the detection rate of US dropped to 34% [9]. The high level of serum AFP can provide the first indication of HCC, which prompting further medical imaging and investigations [10, 11]. However, the sensitivity of AFP in the early detection of HCC is only 39 to 65%, which means that its false-negative rate is 35 to 61% [12–14].

To improve the early diagnosis of HCC, many serum markers of HCC have been identified in the past decades, such as Des-γ-carboxy-prothrombin (DCP), AFP lectin-3 fraction (AFP-L3), glypican-3 (GPC3), Golgi protein 73 (GP73), Dickkopf-1 (DKK1) and so on [15, 16]. DCP has been shown to be a diagnostic serum marker for HCC comparable with AFP [17]. However, only 15 to 30% of patients with early HCC present significant concentration of serum DCP [18]. AFP-L3, an isoform of AFP, displays a specificity of more than 95% for HCC and a sensitivity > 90% for HCC > 5 cm but it can only be detected in 35% of patients with small HCC of < 3 cm, which limited its application in early detection of HCC [19–21]. Though GPC3 and GP73 are recently reported to be better than AFP, their efficacies in the detection of early HCC are still in debate [16, 22, 23]. Shen et al. and we have all reported that Dkk1 display a relative high sensitivity and specificity in the detection of early HCC [24, 25]. However, these results need to be confirmed in more researches. The novel effective serum marker, which is better than AFP or could complement the efficacy of AFP in diagnosing early HCC, are still required [24].

Epidermal growth factor-like domain 7 (Egfl7) is a recently identified secreted protein which binds the extracellular matrix surrounding the blood vessels and it appears to act in an autocrine manner to promote angiogenesis [26–28]. Egfl7 is expressed at high levels in early embryos of mice, but only high expressed in a few organs in adult mice such as the lung, heart and kidney [29]. However, Egfl7 is strongly upregulated in tumors [30]. Previously, we found that Egfl7 was significantly upregulated in HCC tissues and the high expression of Egfl7 protein was closely correlated with clinicopathological parameters (such as vein invasion, multiple nodes and capsule formation) and poor prognosis of HCC [31, 32]. Importantly, we have also demonstrated that Egfl7 could promote the metastasis of HCC by enhancing cell motility through EGFR-dependent FAK (focal adhesion kinase) phosphorylation [31]. As a secreted protein, we subsequently explored the serum levels of Egfl7 in patients with a series of epithelial tumors including HCC as well as healthy individuals, the results documented a significantly higher level of serum Egfl7 in patients with HCC than those patients with other tumors and healthy individuals, shedding a light on the potential application of Egfl7 as a serum diagnostic marker for HCC [32]. However, the efficacy of serum Egfl7 in diagnosing early HCC remains unknown.

Therefore, we carried out the present study to measure the levels of serum Egfl7 in patients with early HCC and evaluate the efficacy of Egfl7 in the diagnosis of early HCC by comparing with AFP.

## Materials and methods

### Serum samples

The Testing Cohort Serum samples were collected from the patients operated for early HCC (*n* = 314) [33], metastatic liver cancer (*n* = 16), benign liver tumors (including cavernous hemangioma of the liver and hepatic adenoma, *n* = 19), and the patients with chronic liver disease (CLD) including cirrhosis and chronic viral hepatitis (*n* = 300) [34, 35] in Xiangya Hospital, Central South University (CSU) from June 2006 to October 2009. At the same time, serum samples of healthy individuals (*n* = 432) were collected from the healthy blood donors of Changsha Blood Bank. The demographic data of these patients and individuals were showed in Table 1. The clinicopathological characteristics for the patients with HCC or CLD were also showed in Table 2 and Table 3 respectively.

**Table 1:** Demographic data for the patients and healthy individuals in Testing and Validation Cohorts

Groups	Validation Cohort							
	***n***	Gender		Age [years, median (range)]	***n***	Gender		Age [years, median (range)]
		Male	Female			Male	Female	
Early HCC	314	275	39	49 (20–78)	158	142	16	50 (22–78)
Chronic Liver Disease	300	222	78	44 (17–83)	120	96	24	43 (17–77)
Metastatic Liver Cancers	16	8	8	59 (23–75)	12	7	5	61 (25–75)
Benign Liver Tumors	19	12	7	49 (14–63)	14	9	5	47 (15–65)
Healthy individuals	432	221	211	22 (18–54)	172	83	89	21 (18–53)

*HCC* Hepatocellular Carcinoma

**Table 2:** Clinicopathological features for HCC patients in Testing and Validation Cohort

Clinicopathological features	Variables	Testing Cohort (***n*** = 314)	Validation Cohort (***n*** = 158)
Etiology	HBV	237	119
	HCV	0	0
	Non-B-non-C	77	39
Liver cirrhosis	Presence	239	125
	Absence	75	33
Serum AFP level	≥ 20 μg/L	194	93
	< 20 μg/L	120	65
Maximal tumor size	≤ 2 cm	61	22
	> 2 cm	253	136
Edmondson-Steiner grade	I – II	168	62
	III – IV	146	96
Capsular formation	Presence	173	77
	Absence	141	81
Tumor nodule number	Solitary	151	80
	Multiple (≤ 3)	163	78
BCLC stage	0	55	21
	A	259	137

*HCC* Hepatocellular carcinoma, *HBV* with hepatitis B virus infection, *HCV* with hepatitis C virus infection, *Non-B-non-C* without hepatitis B or hepatitis C virus infection, *AFP* α-fetoprotein

**Table 3:** Clinicopathological features for patients with chronic liver disease in Testing and Validation Cohort

Clinicopathological features	Variables	Testing Cohort (***n*** = 300)	Validation Cohort (***n*** = 120)
Etiology	HBV	246	93
	HCV	10	4
	Non-B-non-C	58	23
Liver cirrhosis	Presence	190	78
	Absence	110	42
Serum AFP level	≥ 20 μg/L	119	50
	< 20 μg/L	181	70
ALT [IU/mL, media (range)]	N.A.	55.3 (8.1–189.7)	52.8 (13.3–167.5)
AST [IU/mL, media (range)]	N.A.	49.4 (15.5–117.2)	51.6 (25.9–138.8)
Total bilirubin [μmol/L, media (range)]	N.A.	27.3 (15.1–87.9)	25.4 (13.2–83.1)
Child-Pugh Class	A	204	73
	B	85	43
	C	11	4

*HBV* with hepatitis B virus infection, *HCV* with hepatitis C virus infection, *Non-B-non-C* without hepatitis B or hepatitis C virus infection, *AFP* α-fetoprotein, *ALT* Alanine aminotransferase, *AST* Aspartate aminotransferase, *N.A.* not applicable

The Validation Cohort At the end of the first part of the present study, serum samples from a second set of individuals were collected from November 2009 to March 2011 to serve as the validation cohort. This Cohort consisted of healthy individuals (*n* = 172) and patients who were operated for early HCC (*n* = 158) [33], metastatic liver cancer (*n* = 12), benign liver tumors (including cavernous hemangioma of the liver and hepatic adenoma, *n* = 14) or patients with chronic liver disease (*n* = 120) [34, 35]. The demographic and clinical characteristics for these individuals and patients were collected prospectively (Tables 1, 2 and 3).

Early HCC was classified by Barcelona Clinic Liver Cancer (BCLC) criterion as stage 0 ~ A [33]. The diagnosis of HCC was confirmed by histopathological study after hepatic resection. The diagnosis of cirrhosis was based either on histopathology of liver biopsy samples or on the evidence of clinical, laboratory, and imaging examination [24]. And the diagnosis of chronic viral hepatitis was based on the criteria used in the guidelines from American Association for the Study of Liver Diseases (AASLD) [34, 35]. Patients diagnosed with chronic liver disease were limited to those patients who had no history of HCC, there was no ultrasonic evidence of HCC for more than 6 months from the day of serum collection, and their hepatic functions were in the compensated phase. The serum samples were spun, aliquoted and stored at − 80 °C until testing.

Prior informed consent was obtained from the subjects for collection of serum samples in accordance with the guidelines of CSU and the study protocols were approved by the Ethics Committee of CSU.

### Egfl7 and AFP detection

To avoid subjective bias and to reduce system error, a double-blind principle was followed in this study according to the criteria of STARD (standards for reporting of diagnostic accuracy studies) 2015 for reporting studies of diagnostic accuracy [36]. Individuals who collected the serum samples did not participate in the testing and the testing personnel did not know the sources and the groups of the samples. After testing was completed, the information on the samples and the corresponding results were disclosed, and the values of Egfl7 and AFP were analyzed.

Serum Egfl7 levels were measured quantitatively by a sandwich enzyme linked immunosorbent assay (ELISA) system. Firstly, a murine monoclonal antibody (Abnova, Taiwan, China) specific to human Egfl7 protein, as a capture antibody, was added into a 96-well microplate (Greiner bio-one, Germany) and incubated at room temperature for 2 h. Then these 96-well microplates were coated at 4 °C overnight and washed by 0.05% PBST (phosphate buffered saline with Tween-20) to remove any unbound antibody. And 4% BSA (bovine serum albumin) was added into these 96-well microplates for blocking and incubated at 4 °C for 2 h. Secondly, serum samples (250-fold dilution) were added into the 96-well microplates after washing with 0.05% PBST and incubated at room temperature for 2 h. Thirdly, a rabbit polyclonal antibody (Santa Cruz, USA) specific to human Egfl7 protein, as a detection antibody was added into the wells, after washing away any unbound substances with 0.05% PBST, and incubated at room temperature for 2 h. Fourthly, a goat anti-rabbit IgG antibody was added into the wells after washing again with 0.05% PBST and incubated at room temperature for 2 h. After washing with 0.05% PBST, TMB (tetramethyl benzidine) soluble reagent (Tiangen Biotech Co., Ltd., Beijing, China) was added into the wells and incubated to react at room temperature for 30 min. Lastly, H_2_SO_4_ (1 M, Jingmei Biotech Co., Ltd., Beijing, China) was added to stop the reaction and a photometer was employed to determine the intensity of color at a wavelength of 450 nm, with a reference wavelength of 570 nm. We also tested recombinant protein Egfl7 (Abnova, Taiwan, China) in each assay as a standard sample. Serum AFP was detected in the same specimens by using commercially available immunoassays utilizing enhanced chemiluminescence. The upper limit for serum AFP level was set as 1210 ng/mL for any serum AFP levels higher than this value. And the upper normal limit of serum AFP level was set as 20.0 ng/mL.

### Statistical analysis

SPSS 13.0 (Chicago, IL, USA) was employed to perform all the statistical analyses. The comparisons of multi-group for continuous variables were performed by one-way analysis of variance (ANOVA) with Tukey test as post hoc test. For the categorical variables, Chi-square test or Fisher’s exact test (where appropriate) was used. Receiver operating characteristic (ROC) curve was established to get the cut-off value of Egfl7 by using Youden’s index and compare the diagnostic efficacies of serum Egfl7 and AFP. The area under the ROC (AUROC) curves were constructed and compared using the Z test. Correlation between serum Egfl7 and AFP levels was evaluated by the Pearson correlation coefficient. All analyses were two-sided and *P* < 0.05 was considered as statistically significant.

## Results

### Serum Egfl7 levels in patients with early HCC

**Fig. 1:**
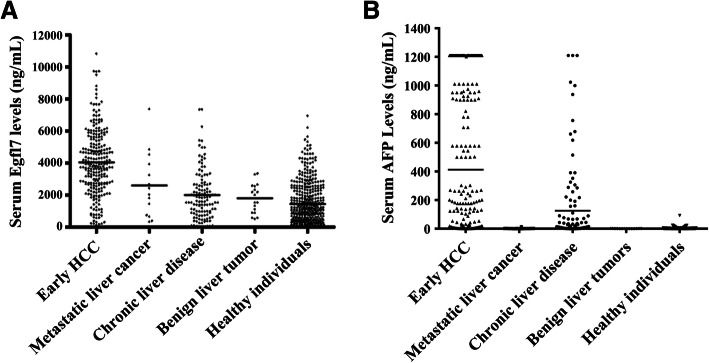
Serum Egfl7 and AFP levels in the Testing Cohort. Serum Egfl7 (**A**) and AFP (**B**) levels in patients from the Testing Cohort with early HCC (*n* = 314), metastatic liver cancer (*n* = 16), benign liver tumors (*n* = 19), chronic liver disease (*n* = 300), and healthy individuals (*n* = 432) were determined by ELISA. The horizontal lines in the figures represented means

**Fig. 2:**
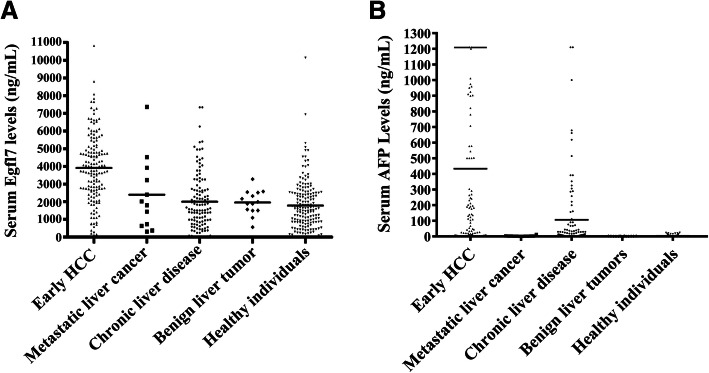
Serum Egfl7 and AFP levels in the Validation Cohort. Serum Egfl7 (**A**) and AFP (**B**) levels in patients from the Validation Cohort with early HCC (*n* = 158), metastatic liver cancer (*n* = 12), benign liver tumors (*n* = 14), or chronic liver disease (*n* = 120), and healthy individuals (*n* = 172) were determined by ELISA. The horizontal lines in the figures represented means

The demographic data of the Testing Cohort and Validation Cohort are shown in Table 1. The serum Egfl7 levels in the Testing Cohort are shown in Fig. 1a. The median serum level of Egfl7 in early HCC was 4017.28 ng/mL (95% CI, 297.6–9271.7 ng/mL), and it was significantly higher than metastatic liver cancer (2707.6 ng/mL; 95% CI, 274.2–5234.8 ng/mL; *P* = 0.0056), benign liver tumors (1756.6 ng/mL; 95% CI, 494.8–3343.8 ng/mL; *P* = 0.0023), chronic liver disease (1600.3 ng/mL; 95% CI, 20.4–6232.1 ng/mL; *P* < 0.0001) and healthy individuals (1082.80 ng/mL; 95% CI, 7.5–4906.1 ng/mL; *P* < 0.0001). Although there was no significant difference between the groups of benign liver tumors and chronic liver disease (*P* = 0.7825), the serum Egfl7 levels in these two groups were higher than the healthy individuals (*P* = 0.0138 and *P* = 0.0161, respectively). Similar results were obtained from the Validation Cohort which also showed that the serum Egfl7 level in early HCC was significantly higher than that in the other groups (Fig. 2a). Serum AFP levels in the Testing Cohort and Validation Cohort were also showed in Fig. 1b and Fig. 2b respectively.

### The diagnostic efficacies of serum Egfl7 in discriminating early HCC from the healthy individuals

ROC curve analyses and the area under the ROC (AUROC) were used to evaluate the diagnostic efficacies of serum Egfl7 with AFP. And the cut-off value for serum Egfl7 was determined as 2610.0 ng/mL by Yoden’s index method.

**Fig. 3:**
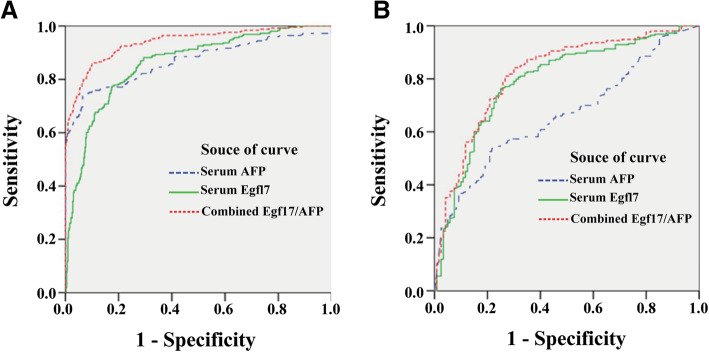
ROC curves of Egfl7, AFP or combined Egfl7 and AFP in the Testing Cohort. ROC curves of Egfl7, AFP or combined Egfl7 and AFP in differentiating early HCC patients from healthy individuals (**A**) or patients with chronic liver disease (**B**) in the Testing Cohort

In the Testing Cohort, Egfl7 showed a significantly higher sensitivity than AFP in discriminating early HCC from healthy individuals (77.4% vs. 65.3%, *P* = 0.0013; Table 2), and the AUROC [0.860 (95% CI: 0.840–0.895) vs. 0.868 (95% CI: 0.851–0.915), *P* = 0.704; Fig. 3a) and accuracy (80.2% vs. 83.8%, *P* = 0.184; Table 4) of Egfl7 were comparable with AFP. However, the specificity of Egfl7 was lower than AFP (82.2% vs. 97.2%, *P* < 0.0001; Table 4).

**Table 4:** Efficacies of serum Egfl7, AFP or Parallel in differentiating early HCC from healthy individuals

Data	Testing Cohort			Validation Cohort		
	Egfl7	AFP	Parallel	Egfl7	AFP	Parallel
Sensitivity	77.4%	65.3%	88.9%	75.9%	58.9%	86.7%
Specificity	82.2%	97.2%	79.9%	79.1%	97.1%	76.2%
Accuracy	80.2%	83.8%	83.6%	77.6%	78.8%	81.2%
Omission diagnostic rate	22.6%	34.7%	11.1%	24.1%	41.1%	13.3%
Mistake diagnostic rate	17.8%	2.8%	20.1%	20.9%	2.9%	23.8%
Positive likelihood ratio	4.34	23.5	4.42	3.63	20.3	3.64
Negative likelihood ratio	0.28	0.36	0.14	0.30	0.42	0.17
Positive predict value	0.76	0.95	0.76	0.77	0.95	0.77
Negative predict value	0.83	0.79	0.91	0.78	0.72	0.86

*HCC* Hepatocellular carcinoma, *AFP* α-fetoprotein, *Parallel* combined Egf17 and AFP

**Fig. 4:**
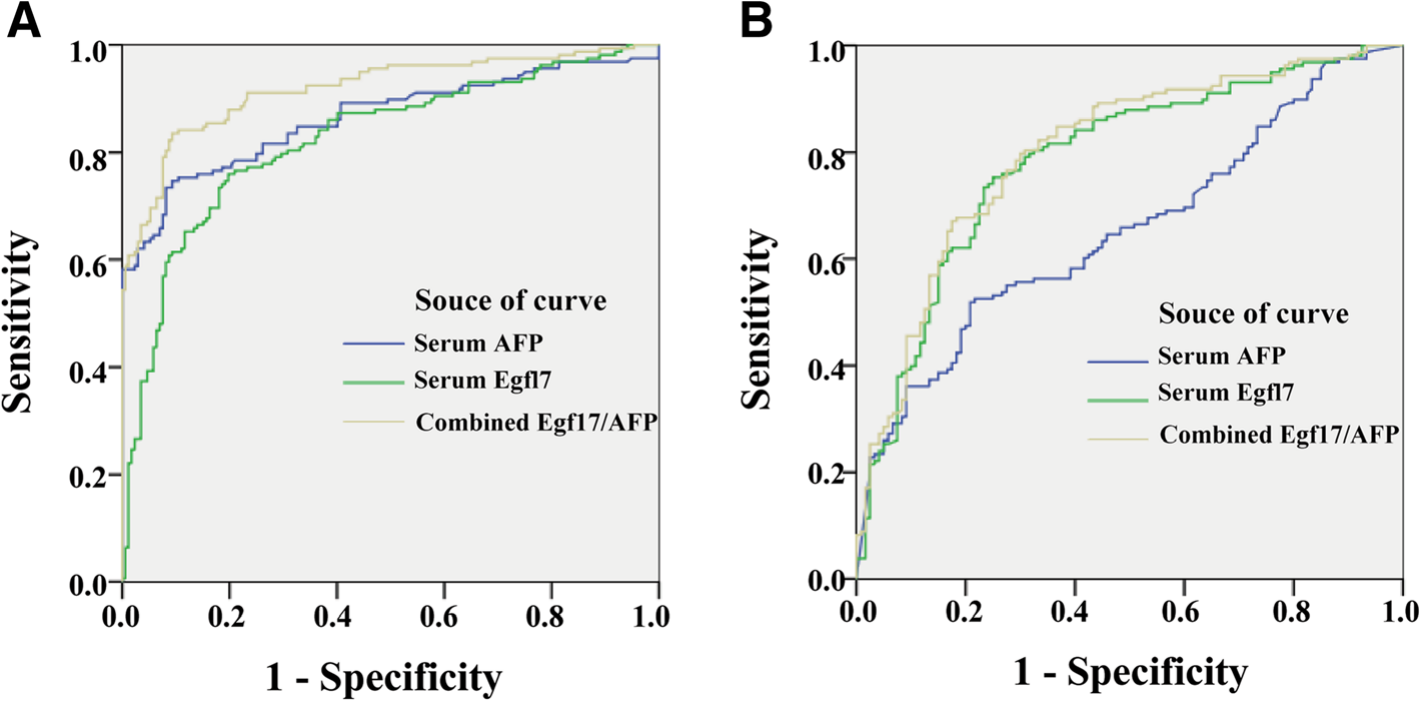
ROC curves of Egfl7, AFP or combined Egfl7 and AFP in the Validation Cohort. ROC curves of Egfl7, AFP or combined Egfl7 and AFP in differentiating early HCC patients from healthy individuals (**A**) or patients with chronic liver disease (**B**) in the Validation Cohort

In the Validation Cohort, Egfl7 also showed a distinctly higher sensitivity than AFP (75.9% vs. 58.9%, *P* < 0.0001). Though the specificity of Egfl7 was lower than AFP (79.1% vs. 97.1%, *P* = 0.0007), their accuracy and AUROC were comparable [77.6% vs. 78.8%, *P* = 0.416; 0.827 (95% CI: 0.803–0.855) vs. 0.861 (95% CI: 0.847–0.889), *P* = 0.651; Fig. 4a and Table 4].

### The diagnostic efficacies of serum Egfl7 in differentiating early HCC from the patients with CLD

As the majority of patients with HCC (> 85%) had chronic liver disease (CLD) such as chronic viral hepatitis and/or cirrhosis, the diagnostic value of Egfl7 in the discrimination of early HCC from CLD was further investigated. The results obtained from the Testing Cohort showed that Egfl7 was superior to AFP in full-scale with a significantly higher AUROC [0.800 (95% CI: 0.772–0.828) vs. 0.675 (95% CI: 0.640–0.715), *P* < 0.0001; Fig. 3b], sensitivity (75.2% vs. 61.8%, *P* < 0.0001), specificity (71.7% vs. 62.0%, *P* = 0.0015), accuracy (73.5% vs. 61.9%, *P* < 0.0001), positive likelihood ratio (2.65 vs. 1.56, *P* = 0.0023) and positive predict value (0.74 vs. 0.66, *P* = 0.0074) than AFP (Table 5). In the Validation Cohort, the AUROC, sensitivity and accuracy of Egfl7 in discriminating early HCC from CLD were all dramatically higher than AFP [0.787 (95% CI: 0.717–0.851) vs. 0.563 (95% CI: 0.489–0.648), 75.9% vs. 36.1, 74.1% vs. 59.7%, respectively; *P* < 0.0001; Fig. 4b and Table 5].

**Table 5:** Efficacies of serum Egfl7, AFP or Parallel in discriminating early HCC from chronic liver disease

Data	Testing Cohort			Validation Cohort		
	Egfl7	AFP	Parallel	Egfl7	AFP	Parallel
Sensitivity	75.2%	61.8%	88.9%	75.9%	36.1%	82.9%
Specificity	71.7%	62.0%	60.7%	71.7%	90.8%	66.7%
Accuracy	73.5%	61.9%	75.1%	74.1%	59.7%	75.9%
Omission diagnostic rate	24.8%	38.2%	11.1%	24.1%	63.9%	17.1%
Mistake diagnostic rate	28.3%	39.7%	39.3%	29.3%	9.2%	33.3%
Positive likelihood ratio	2.65	1.56	2.26	2.59	3.92	2.49
Negative likelihood ratio	0.32	0.62	0.18	0.34	0.70	0.26
Positive predict value	0.74	0.66	0.70	0.78	0.84	0.77
Negative predict value	0.73	0.61	0.84	0.69	0.52	0.75

*HCC* Hepatocellular carcinoma, *AFP* α-fetoprotein, *Parallel* combined Egf17 and AFP

### The diagnostic efficacies of serum Egfl7 in distinguishing early HCC from non-HCC individuals

To fully evaluate the diagnostic value of Egfl7 in the early detection of HCC, we pooled the healthy individuals and the patients with CLD into a single control group named as the non-HCC individuals and investigated the efficacies of Egfl7 in distinguishing the patients with early HCC from these individuals. Our results showed that the diagnostic efficacies of Egfl7 was much better than AFP, with a significantly higher sensitivity (76.6% vs. 64%, *P* = 0.0031), specificity (82.7% vs. 68%, *P* < 0.0001), accuracy (79.9% vs. 66.1%, *P* < 0.0001), positive likelihood ratio (4.44 vs. 2.0, *P* = 0.0045) and positive predictive value (0.80 vs. 0.64, *P* = 0.0011) (Table 6). Moreover, the sensitivity and accuracy of Egfl7 in distinguishing early HCC patients from the non-HCC individuals were also better than AFP in the Validation Cohort (75.9% vs. 58.9%, *P* < 0.0001; 76.0% vs. 73.3%, *P* = 0.024; Table 6).

**Table 6:** Efficacies of serum Egfl7, AFP or Parallel in distinguishing early HCC patients from non-HCC individuals

Data	Testing Cohort			Validation Cohort		
	Egfl7	AFP	Parallel	Egfl7	AFP	Parallel
Sensitivity	76.6%	64%	91%	75.9%	58.9%	86.7%
Specificity	82.7%	68%	66%	76.0%	81.2%	62.3%
Accuracy	79.9%	66.1%	77.6%	76.0%	73.3%	70.9%
Omission diagnostic rate	23.4%	36%	9%	24.1%	41.1%	13.3%
Mistake diagnostic rate	17.3%	32%	34%	24.0%	18.8%	37.7%
Positive likelihood ratio	4.44	2.0	2.6	3.16	3.13	2.30
Negative likelihood ratio	0.28	0.53	0.14	0.32	0.51	0.21
Positive predict value	0.80	0.64	0.70	0.63	0.63	0.55
Negative predict value	0.79	0.68	0.89	0.85	0.78	0.90

*HCC* Hepatocellular carcinoma, *AFP* α-fetoprotein, *Parallel* combined Egf17 and AFP

### The efficacy of combined Egfl7 and AFP in the diagnosis of early HCC

The high cut-off value of AFP, such as 100 ng/mL or even 400 ng/mL, was often used in distinguishing the patients with HCC from those with CLD to assure a satisfactory specificity [37, 38]. We also evaluated the efficacies of AFP at different cut-off values for the diagnosis of early HCC from healthy individuals or patients with CLD. When the cut-off values of AFP increased from 20 ng/ml to 400 ng/ml, the sensitivity of AFP was dramatically decreased from 61.8–65.3% to 38.4% (Table 7). More importantly, 70.8% (85 of 120) of early HCC with negative AFP in the Testing Cohort and 67.7% (44 of 65) of AFP-negative early HCC in the Validation Cohort could be diagnosed by Egfl7 (Table 8).

**Table 7:** Efficacies of serum AFP testing at different cutoff value in diagnosing early HCC

Group	Accuracy	Sensitivity	Specificity
**Early HCC vs. healthy individuals**			
**Serum AFP**			
Cut-off value: 20.0 ng/mL	83.8%	65.3%	97.2%
Cut-off value: 100.0 ng/mL	89.1%	55.1%	100%
Cut-off value: 400.0 ng/mL	85.1%	38.4%	100%
**Early HCC vs. chronic liver disease**			
**Serum AFP**			
Cut-off value: 20.0 ng/mL	61.9%	61.8%	62%
Cut-off value: 100.0 ng/mL	65.9%	55.1%	78.3%
Cut-off value: 400.0 ng/mL	62.8%	38.4%	90.8%

*HCC* Hepatocellular carcinoma, *AFP* α-fetoprotein, *Parallel* combined Egf17 and AFP

**Table 8:** Positive rates of serum Egfl7 in early HCC with negative AFP

Testing Cohort				Validation Cohort			
Egfl7	AFP		Total	Egfl7	AFP		Total
	Positive	Negative			Positive	Negative	
Positive	151	85	236	Positive	76	44	120
Negative	43	35	78	Negative	17	21	38
Total	194	120	314	Total	93	65	158

*HCC* Hepatocellular carcinoma, *AFP* α-fetoprotein

Furthermore, there was no significant correlation between the serum Egfl7 and AFP levels (Spearman rank correlation coefficient: *r* = 0.154, *P* = 0.396) and their diagnosis results were not identical (kappa = 0.147, *P* = 0.207), suggesting Egfl7 and AFP combination could be helpful to improve the efficacy of early HCC diagnosis. Consist with the hypothesis, in differentiating the patients with early HCC from healthy individuals or patients with CLD, the combination significantly improved the sensitivity to 88.9% and increase the AUROC to 0.967 (95% CI: 0.940–0.995) or 0.819 (95% CI: 0.796–0.843) (Tables 4 & 5, Figs. 3 & 4) in the Testing Cohort. The combined use of Egfl7 and AFP also could improve the sensitivity to 86.7% or 82.9% in the Validation Cohort, which was remarkably higher than Egfl7 or AFP alone (Tables 4 & 5). In distinguishing early HCC from non-HCC individuals, the combination of Egfl7 and AFP also could improve the sensitivity to 91% (in the Testing Cohort) or 86.7% (in the Validation Cohort) (Table 6).

## Discussion

Since HCC is usually asymptomatic for much of its natural history, most of HCC patients were detected at an intermediate or advanced stage, by which time surgical or oncological treatment options were limited [39]. Therefore, screening and diagnose HCC as early as possible, when it is curable, was critical important to improve the treatment of this deadly disease [6]. For this reason, surveillance in patients with high risks of developing HCC has been recommended, and US and serum AFP are commonly used [5]. Recently, studies have shown that surveillance program improved prognosis of HCC [40]. However, US examination depends on the experience of the sonographer and the technical quality of the US equipment, and is therefore subjective and nonrepetitive [41]. Furthermore, the use of AFP to screen a population with chronic liver disease who is at risk of developing HCC has been reported to have a poor sensitivity of only 20 to 30% at cutoff values > 100 ng/ml [42]. A better screening program is in urgent need for early HCC diagnosis.

Many novel serum diagnostic markers of HCC have been found, including DCP, GPC3, AFP-L3 and GP73 [15]. Although many non-protein serum markers have been identified for HCC in the past decade such as mutated DNAs, methylated DNAs and RNAs [including microRNA, lncRNA (long non-coding RNA) and circRNA (circular RNA) or even these RNAs inside exosomes] [43–47], protein markers could be detected in serum are the most applicable for clinical routine assessments [22, 48], for their advantages including non-invasive, requiring less than 100 μL serum, low dependence on operator expertise, low cost, high reproducibility, and no samples need pretreatment (such as extraction, purification or reverse transcription) [22]. Egfl7, a recently identified protein involved in the progression of HCC, maybe a satisfactory marker for HCC: it is a secretory protein, is specifically overexpressed in HCC cells instead of normal cells such as vascular endothelial cells or cholangiocytes within HCC tissues, and is hardly detectable in human adult normal liver tissues [31, 32]. More importantly, Egfl7 has already been evidenced to be upregulated in the serum of patients with HCC or other type of cancers [32, 49–51], suggesting its possible application in the diagnosis of HCC. However, the levels of serum Egfl7 in patients with early HCC are still unknown.

In the present study, we detected the serum levels of Egfl7 in the patients with early HCC. The results showed a significantly higher level of serum Egfl7 in these patients than healthy individuals (increased by 3.71 folds), which was consist with the elevated levels of serum Egfl7 in HCC patients [32, 49]. In differentiating early HCC from the healthy individuals, Egfl7 had a significantly higher sensitivity than AFP (75.9–77.4% vs. 58.9–65.3%%) and their accuracy and AUROC were similar indicating a generally superior of Egfl7 in the detection of early HCC to AFP as well as some other serum marker for early HCC, such as GPC3 (sensitivity ranged from 47.9 to 66.2%) [52] and DKK1 (sensitivity ranged from 70.9 to 73.8%) [24, 25].

Our results showed serum Egfl7 levels was modestly elevated in patients with CLD compared with healthy individuals (1600.3 ng/mL vs. 1082.80 ng/mL) but much lower than those with early HCC (4017.28 ng/mL). Furthermore, in surveillance of early HCC from CLD, Egfl7 had a much better AUROC (0.787–0.800 vs. 0.563*–*0.675), sensitivity (75.2–75.9% vs. 36.1–61.8%) and accuracy (73.5–74.1% vs. 59.7–61.9%) than AFP, indicating Egfl7 as a better serum marker than AFP in the surveillance of early HCC from CLD. Moreover, Egfl7 also exhibited an advantage in the aspect of sensitivity compared with some recently reported serum markers for distinguishing early HCC from CLD such as GPC3 (sensitivity of 55%) [22], GP73 (sensitivity of 62%) [53] and DKK1 (sensitivity ranged from 54.8 to 73.8%) [24, 25].

Although AFP is often recommended as a serum marker for the surveillance of HCC, the use of AFP to screen a population with chronic liver disease who is at risk of developing HCC has been reported to have a poor sensitivity of only 20 to 30% at cutoff values > 100 ng/mL [42]. In this study, to distinguish early HCC from chronic liver disease, AFP had a specificity which ranged from 62 to 90.8% when the cut-off value increased from 20 ng/mL to 400 ng/mL but the sensitivity correspondingly decreased from 61.8 to 38.4%, which meaning 61.6% of early HCC was missed at a cut-off value of 400 ng/mL. Therefore, the recognition of AFP-negative HCC is important to improve the efficacy of early detection of HCC. In the present study, 67.7–70.8% of patients with early HCC who had a negative AFP could be diagnosed by Egfl7, which was slightly better than AFP-L3 (50%) [54], GP73 (66–67%) [16, 54] and comparable with DKK1 (67.3–73.1%) [24, 25]. In consideration of a high specificity of AFP in the surveillance and early detection of HCC, Egfl7 might be helpful to make up the deficiency of AFP in sensitivity and further improve the diagnostic efficacy of early HCC. The combination of Egfl7 and AFP showed a significantly increased sensitivity ranged from 88.9 to 91.0%, which was not only remarkably higher than the sensitivity of Egfl7 or AFP alone but also the sensitivity of AFP combined with other serum markers such as DKK1 (63.8–90.8%) [24, 25] and GPC3 (81–89%) [22].

In addition, we have found that the level of serum Egfl7 was also elevated in patients with metastatic liver cancer (increased by 2.50 folds) or benign liver tumors (increased by 1.62 folds) also had a moderately elevated compared with the healthy individuals, although the magnitude is smaller than that in early HCC (3.71 folds), suggesting serum Egfl7 might be helpful to determine the nature (benign tumor or HCC) and origin (primary or secondary) of liver tumors.

To our knowledge, this is the first large-scale study to report the performance of Egfl7 as a serum diagnostic marker for early HCC in a test cohort and an independent validation cohort. The results indicate Egfl7 as a novel and effective serological marker for the early detection of HCC with a better sensitivity and accuracy than AFP, which also could improve the efficacy of AFP, especially in the surveillance of a high-risk population with CLD and identification of the early HCC patients with negative AFP.

## Data Availability

All data generated or analysed during this study are included in this published article.

## Abbreviations

AASLD: American association for the study of liver diseases
AFP: Alpha-fetoprotein
AFP-L3: *Lens culinaris* agglutinin-reactive fraction of alpha- fetoprotein
AUROC: Area under the ROC curve
BCLC: Barcelona clinic liver cancer criterion
BSA: Bovine serum albumin
CI: Confidence interval
circRNA: circular RNA
CLD: Chronic liver disease
CSU: Central south university
DCP: Des-gamma-carboxy prothrombin
DKK1: Dickkopf-1
Egfl7: Epidermal growth factor-like domain 7
ELISA: Enzyme linked immunosorbent assay
FAK: Focal adhesion kinase
GP73: Golgi protein 73
GPC3: Glypican-3
HCC: Hepatocellular carcinoma
lncRNA: long non-coding RNA
PBST: Phosphate buffered saline with Tween-20
ROC: Receiver operating curve
STARD: Standards for reporting of diagnostic accuracy studies
TMB: Tetramethyl benzidine
US: Ultrasound
